# Molecular docking and dynamics simulation studies uncover the host-pathogen protein-protein interactions in *Penaeus vannamei* and *Vibrio parahaemolyticus*

**DOI:** 10.1371/journal.pone.0297759

**Published:** 2024-01-24

**Authors:** Nur Fathiah Rosilan, Muhamad Arif Mohamad Jamali, Siti Aishah Sufira, Khor Waiho, Hanafiah Fazhan, Noraznawati Ismail, Yeong Yik Sung, Zeti-Azura Mohamed-Hussein, Azzmer Azzar Abdul Hamid, Nor Afiqah-Aleng

**Affiliations:** 1 Institute of Climate Adaptation and Marine Biotechnology (ICAMB), Universiti Malaysia Terengganu, Kuala Nerus, Terengganu, Malaysia; 2 Faculty of Science and Technology, Universiti Sains Islam Malaysia, Bandar Baru Nilai, Nilai, Negeri Sembilan, Malaysia; 3 Research Unit for Bioinformatics and Computational Biology (RUBIC), Kuliyyah of Science, International Islamic University Malaysia, Bandar Indera Mahkota, Kuantan, Pahang, Malaysia; 4 Higher Institution Centre of Excellence (HICoE), Institute of Tropical Aquaculture and Fisheries, Universiti Malaysia Terengganu, Kuala Nerus, Terengganu, Malaysia; 5 Centre for Chemical Biology, Universiti Sains Malaysia, Minden, Penang, Malaysia; 6 UKM Medical Molecular Biology Institute, UKM Medical Centre, Jalan Yaacob Latiff, Cheras, Kuala Lumpur, Malaysia; 7 Department of Applied Physics, Faculty of Science and Technology, Universiti Kebangsaan Malaysia, UKM Bangi, Selangor, Malaysia; CIFRI: Central Inland Fisheries Research Institute, INDIA

## Abstract

Shrimp aquaculture contributes significantly to global economic growth, and the whiteleg shrimp, *Penaeus vannamei*, is a leading species in this industry. However, *Vibrio parahaemolyticus* infection poses a major challenge in ensuring the success of *P*. *vannamei* aquaculture. Despite its significance in this industry, the biological knowledge of its pathogenesis remains unclear. Hence, this study was conducted to identify the interaction sites and binding affinity between several immune-related proteins of *P*. *vannamei* with *V*. *parahaemolyticus* proteins associated with virulence factors. Potential interaction sites and the binding affinity between host and pathogen proteins were identified using molecular docking and dynamics (MD) simulation. The *P*. *vannamei-V*. *parahaemolyticus* protein-protein interaction of Complex 1 (Ferritin-HrpE/YscL family type III secretion apparatus protein), Complex 2 (Protein kinase domain-containing protein-Chemotaxis CheY protein), and Complex 3 (GPCR-Chemotaxis CheY protein) was found to interact with -4319.76, -5271.39, and -4725.57 of the docked score and the formation of intermolecular bonds at several interacting residues. The docked scores of Complex 1, Complex 2, and Complex 3 were validated using MD simulation analysis, which revealed these complexes greatly contribute to the interactions between *P*. *vannamei* and *V*. *parahaemolyticus* proteins, with binding free energies of -22.50 kJ/mol, -30.20 kJ/mol, and -26.27 kJ/mol, respectively. This finding illustrates the capability of computational approaches to search for molecular binding sites between host and pathogen, which could increase the knowledge of *Vibrio* spp. infection on shrimps, which then can be used to assist in the development of effective treatment.

## Introduction

Shrimp aquaculture is a rapidly growing animal food-producing sector because it is in high demand and contributes significantly to the global economy [1, 2]. Among various cultured shrimp species, the whiteleg shrimp, *Penaeus vannamei*, is the most commonly cultured species worldwide, with a reported production of 6.3 million tonnes in 2021 [3]. However, bacterial infection has led to a high mortality rate, and it has become one of the main constraints to the success of shrimp aquaculture, which often results in a significant global economic loss [4]. *Vibrio parahaemolyticus*, a gram-negative and rod-shaped bacteria [5], is one of the most common marine bacteria that infect *P*. *vannamei*. It can potentially cause disease outbreaks, resulting in mass mortality in cultured shrimps [6]. One of the prevalent diseases caused by *V*. *parahaemolyticus* is acute hepatopancreatic necrosis disease (AHPND), causing the infected shrimps to be lethargic and exhibit stunted growth. Ultimately, this infection is able to cause 100% mortalities within 30 to 40 days after stocking [7, 8]. In addition to AHPND, *V*. *parahaemolyticus* can also cause white faeces disease (WFD) in the infected *P*. *vannamei* [9]. WFD is characterised by the presence of white faecal strings and golden brown intestines among infected individuals [10]. WFD causes mass mortalities after approximately 50 days of culture, reducing the survival rate by 20% to 30% among infected individuals [11]. *V*. *parahaemolyticus* is also able to cause glass post-larvae disease (GPD) in *P*. *vannamei*, with more than 90% of the mortality observed between 24 to 48 hours after the first appearance of abnormal symptoms among the infected shrimps, such as pale hepatopancreas and a colourless digestive tract [12, 13].

The pathogenicity of *V*. *parahaemolyticus* can be determined with the presence of virulence factors, which are specific molecules produced by the bacteria that assist in host colonisation [14]. One of the common virulence factors in *V*. *parahaemolyticus* is the type III secretion system (T3SS) [15]. T3SS is a needle-like bacterial structure known as an injectisome that permits the gram-negative bacteria to inject and secrete effector proteins into the cytosol of *P*. *vannamei* [16]. T3SS consists of T3SS1 and T3SS2, located in chromosome 1 and chromosome 2, respectively [17], and both of them mediate toxin secretion, leading to cytotoxicity and enterotoxicity in *P*. *vannamei* during infection [18]. Besides T3SS, flagella is another important virulence factor in *V*. *parahaemolyticus* that provides motility and confers various interactions such as invasion, adhesion, and biofilm formation between bacteria and the hosts [19, 20]. These virulence factors may attack immune-related proteins to suppress the host immune systems, which then potentially leads to disease development [21]. Therefore, studying specific interactions between *V*. *parahaemolyticus* proteins involved in virulence and *P*. *vannamei* immune-related proteins may enhance the knowledge of bacteria infection in shrimps.

Molecular docking, specifically protein-protein docking, followed by molecular dynamics (MD) simulation, are valuable computational approaches that can be used to study protein-protein interaction (PPI). Protein-protein docking predicts possible binding sites between two protein structures [22], while MD simulations are used to validate the prediction of protein-protein docking and its binding affinity [23]. Protein-protein docking prediction and MD simulations are used to reveal potential mechanisms of pathogen entry into shrimp and identify the interacting residues involved in infection [24–26]. Ji et al. [26] used this approach to identify the binding of white spot syndrome virus (WSSV) envelope protein VP28 to the secondary beta-sheet of Rab7 residues of *P*. *chinensis* at LEU73 to ASP86. They also predicted GLU81, PHE77, and ASP76 of *Pc*Rab7 as possible interaction sites for the *Pc*Rab7-VP28 complex, suggesting their potential roles in WSSV infection of *P*. *chinensis* [26]. Similarly, the possible interaction sites for WSSV and Rab7 protein of *P*. *monodon* were predicted at ARG69-SER74, VAL75-ILE143, LEU73-ILE143, ARG79-ASN144, and ALA198-ALA182, and these interaction sites contributed to the formation of *Pm*Rab7-VP28 complex [24]. Chitin-binding protein (CBP) binds to envelop protein VP24 of WSSV *via* the interactions between GLY1-TYR5, THR5-HIS4, ASP88-ASN2, and CYS86-LEU7, which suggested the potential WSSV entry mechanism in *P*. *monodon* [25]. These findings illustrate the ability of protein-protein docking and MD simulation to determine specific points of interactions between host and pathogen proteins and identify binding modes and the dynamics of protein complexes.

Rosilan et al. [27] have conducted a host-pathogen PPI (HP-PPI) network analysis between *P*. *vannamei* and *V*. *parahaemolyticus* using interolog- and domain-based methods and several pairs of interactions that might be involved in *V*. *parahaemolyticus* infection in *P*. *vannamei* were identified. Those interactions are identified between ferritin-HrpE/YscL family type III secretion apparatus protein, protein kinase domain-containing protein-chemotaxis CheY protein, and G protein-coupled receptor kinase (GPCR)-chemotaxis CheY protein [27]. This study aims to elucidate the above-mentioned interactions by determining the binding sites and evaluating their affinity using structure-based approaches such as protein-protein docking and MD simulation. The binding sites on the host (*P*. *vannamei*) and pathogen (*V*. *parahaemolyticus*) proteins and their binding affinities are highlighted, and the information will provide an in-depth understanding of the molecular interactions between host and pathogen, which may be useful in future studies to improve shrimp aquaculture production, such as developing strategies to impede or prevent pathogen entry into the shrimp immune system.

## Computational methods

### Retrieval of three-dimensional (3D) models

The protein sequence of *P*. *vannamei* (ferritin–A0A423SK59; protein kinase domain-containing protein–A0A423SQ07; G protein-coupled receptor kinase (GPCR)–A0A423TB58) and *V*. *parahaemolyticus* (HrpE/YscL family type III secretion apparatus protein–A0A6H0JL36; Chemotaxis CheY protein–Q79YX1) were retrieved from UniProt database (https://www.uniprot.org/) [28]. The three-dimensional (3D) structure model of respected proteins were retrieved from the AlphaFold protein structure database (https://alphafold.ebi.ac.uk/) [29, 30]. AlphaFold is an artificial intelligence (AI) system developed by DeepMind, has been ranked as a top structure prediction method in Critical Assessment of Structure Prediction 14 (CASP14) [30] due to its ability to accurately predict 3D model of a protein from its amino acids. The information on each protein structure, such as alpha (α)-helices, beta (β)-sheets, domain, and two-dimensional (2D) topology diagram, was retrieved from PDBsum (http://www.ebi.ac.uk/thornton-srv/databases/pdbsum/) [31]. PDBsum is a web server that provides 3D structure information such as protein domains, interactions, secondary structures, PROCHECK analysis, enzyme reaction, and domain architecture [31]. The quality of the 3D protein structure of *P*. *vannamei* and *V*. *parahaemolyticus* was validated to predict the reliability of the retrieval 3D protein structure using ERRAT (https://saves.mbi.ucla.edu/) [32], PROCHECK (https://saves.mbi.ucla.edu/) [33, 34], MolProbity (http://molprobity.biochem.duke.edu/index.php) [35], protein quality predictor (ProQ) (https://proq.bioinfo.se/cgi-bin/ProQ/ProQ.cgi) [36], and protein structure analysis (ProSA) (https://prosa.services.came.sbg.ac.at/prosa.php) [37, 38].

### Protein-protein docking of *Penaeus vannamei—Vibrio parahaemolyticus* complex

In protein-protein docking, possible binding sites between the protein complexes, i.e. ferritin-HrpE/YscL family type III secretion apparatus protein (Complex 1), protein kinase domain-containing protein-chemotaxis CheY protein (Complex 2), and GPCR-chemotaxis CheY protein (Complex 3) were determined using HawkDock server (http://cadd.zju.edu.cn/hawkdock/) [39–41]. HawkDock uses ATTRACT docking algorithm to analyse PPI and identify key docking residues involved in binding complexes. The HawkRank scoring function determines the docked score and MM/GBSA for free energy decomposition analysis based on weighted energy terms, such as van der Waals potentials, electrostatic potentials, and desolvation potentials [40]. HawkDock generates top ten docked proteins, and the best-docked model is selected based on the lowest docked score [42]. The best-docked model was visualised, and the hydrogen bond (H-bond) was identified within 5 angstroms (Å) of *P*. *vannamei* and *V*. *parahaemolyticus* protein using PyMOL (https://pymol.org/2/) [43]. The best-docked model was then submitted to the PDBsum server to obtain the total and type of other interactions, i.e., salt bridges and non-bonded contacts between protein residues.

### Molecular dynamics (MD) simulation of protein complexes

MD simulation was conducted on the best-docked models using GROMACS software (https://www.gromacs.org/) [44, 45] to evaluate the protein complexes’ binding stability *via* Optimised Potential for Liquid Simulations (OPLS) force fields for each protein complex [46]. MD simulation run was completed after the completion of three stages, i.e., (i) system neutralisation, (ii) system energy minimisation, and (iii) system equilibration. Each protein complex or system was solvated using the spc216 water model in a cubic box of 1.0 nanometer (nm). Sodium ion (NA^+^) was added to neutralise the system for energy minimisation. The steepest descent minimisation algorithm was applied to set up the system energy minimisation with the maximum force below 1000 kJ/mol of 50,000 maximum steps. The systems equilibration was performed on 50 picoseconds (ps) using the Particles Mesh Ewald (PME) electrostatics approach to compute long-range electrostatics, contributing to reliable energy estimates [47]. The 100 nanoseconds (ns) MD simulations were conducted in triplicate by collecting data every 100 ps after the systems were equilibrated at a constant temperature of 300 Kelvin (K). The trajectory file of root-mean-square deviation (RMSD), the radius of gyration (R_g_), H-bond, and distance was analysed by computing the average from triplicate MD simulation runs to measure the binding affinity between protein complexes. Pre- and post-MD structures were superimposed to observe conformational changes, structural stability, and PPI. Several tools were utilised to analyse the MD simulation results, such as visual molecular dynamics (VMD) (https://www.ks.uiuc.edu/Research/vmd/) [48] for displaying and animating MD simulation trajectories using 3D graphics, Xmgrace (https://plasma-gate.weizmann.ac.il/Grace/) [49] for plotting the data of RMSD, R_g_, H-bond, and distance and PyMOL to visualise the extracted 3D structures of complex after MD simulation.

### Principal component analysis (PCA)

The collective motion of each protein complex from the MD simulation trajectories was analysed using principal component analysis (PCA) over 100 ns in triplicate MD simulation using GROMACS [44]. PCA was conducted by creating the covariance matrix of backbones atoms using the gmx covar tool. The first two principal components, PC1 (projection on eigenvector 1) and PC2 (projection of eigenvector 2), were analysed using the gmx anaeig tool to observe the global motion of Complex 1, Complex 2, and Complex 3. The results of PCA (PC1 and PC2) were visualised using the scatter plot for each replicate.

### Cluster analysis

The most representative protein conformation throughout the 100 ns simulation was determined by further analysing the MD simulation trajectories of each complex through clustering analysis using the Gromos clustering algorithm [50] in GROMACS. The trajectory file (.xtc) of each replicate was combined for each complex using the gmx trjcat module before clustering. The gmx cluster module was used to cluster the MD simulation trajectories based on 0.2 nm RMSD cut-off values. The .pdb file of top cluster coordinates (first coordinates, middle coordinates, last coordinates) for each complex was extracted from MD simulation trajectories. The interacting residues involved in *P*. *vannamei*-*V*. *parahaemolyticus* PPI of each coordinate was retrieved by PDBsum to compare the interactions and visualised using PyMOL.

### Molecular mechanics poisson-boltzmann surface area (MM/PBSA) calculation

**Fig 1:**
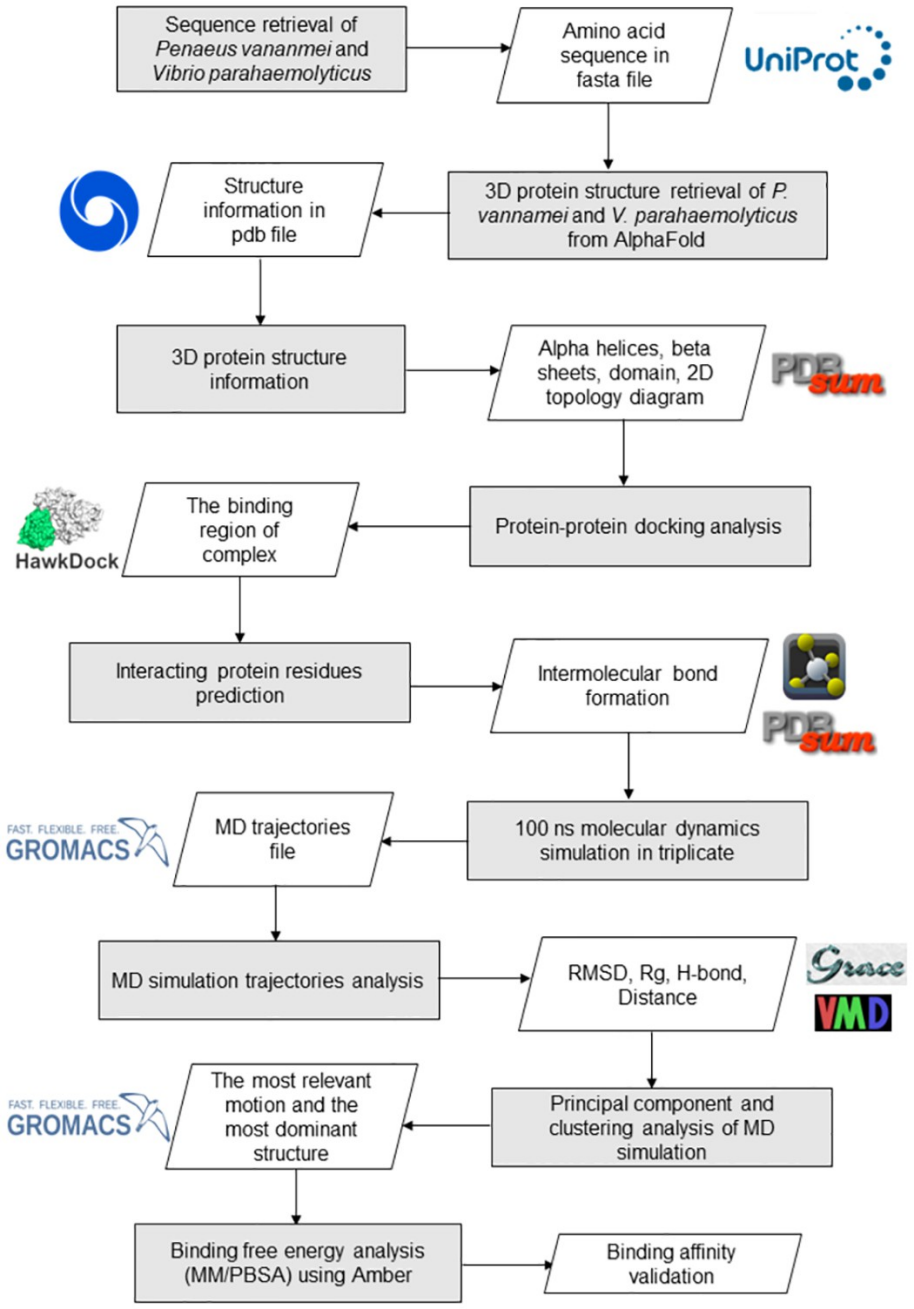
Workflow of the study. The rectangle box represents the method. The parallelogram indicates the output.

Molecular Mechanics Poisson-Boltzmann surface area (MM/PBSA) approach was used to calculate the binding free energy of several energy components, i.e., (i) van der Waals force (VDWAALS), (ii) electrostatic energy (EEL), (iii) the electrostatic contribution to the solvation free energy (EGB), and (iv) non-polar contribution to the solvation free energy (ESURF) between *P*. *vannamei* and *V*. *parahaemolyticus* proteins. Kollman et al. [51] developed MM-PBSA as the end-point for free energy methods as it is more efficient and accurate than scoring functions and computationally inexpensive [52]. The binding free energy analysis was calculated using the MM-PBSA.py tool in Amber (https://ambermd.org/) [53]. The overall workflow is illustrated in Fig 1.

## Results

### Predicted structure of *Penaeus vannamei* and *Vibrio parahaemolyticus* proteins

**Fig 2:**
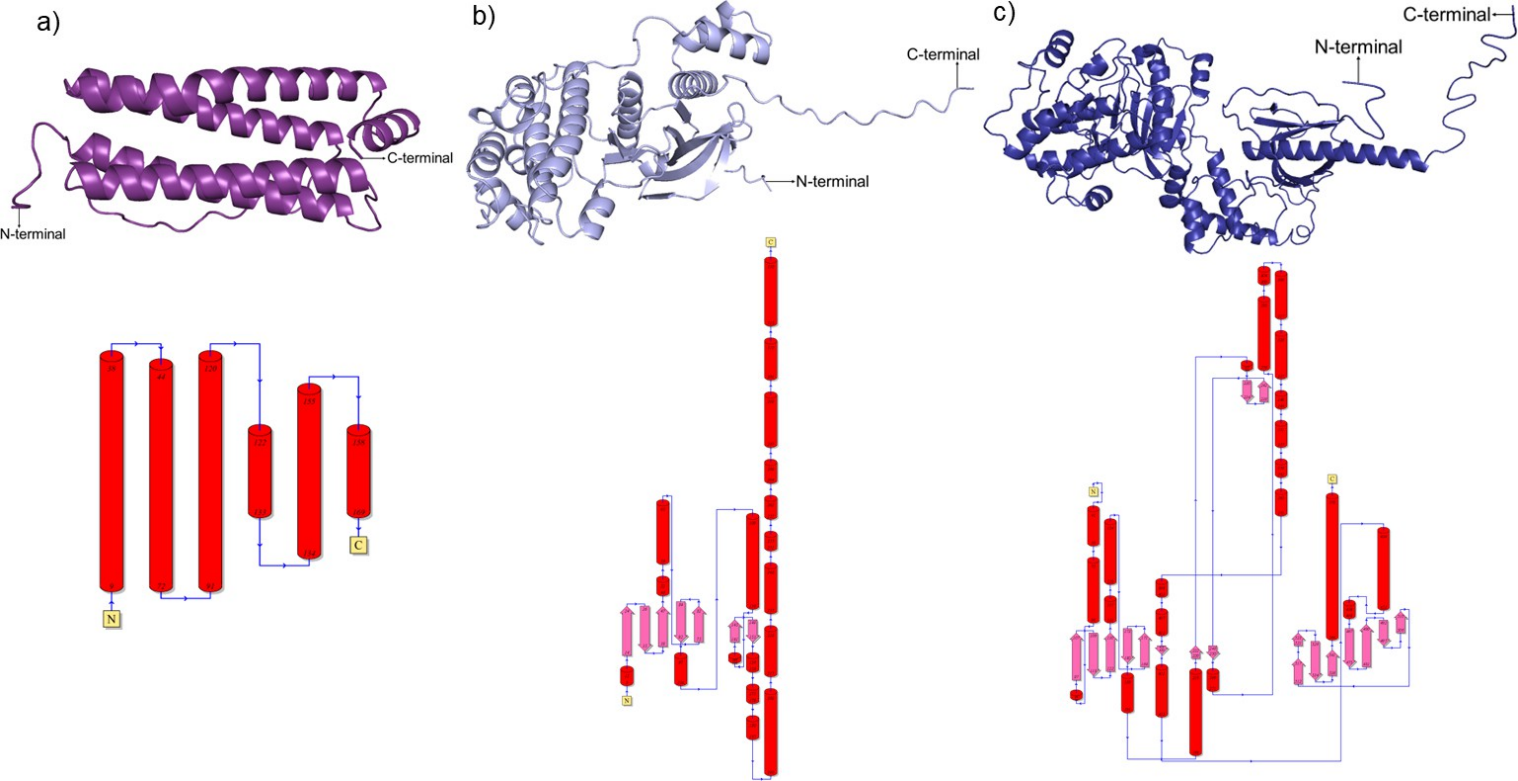
3D models and 2D topology diagrams of *Penaeus vannamei* proteins. (a) Ferritin (A0A423SK59), (b) Protein kinase domain-containing protein (A0A423SQ07), (c) G protein-coupled receptor (A0A423TB58). The red cylinder in the topology diagram represents α-helix, and the pink arrow indicates the β-strand to form β-sheet. A small blue arrow illustrates the direction of the protein chain from N- to C-terminal.

**Fig 3:**
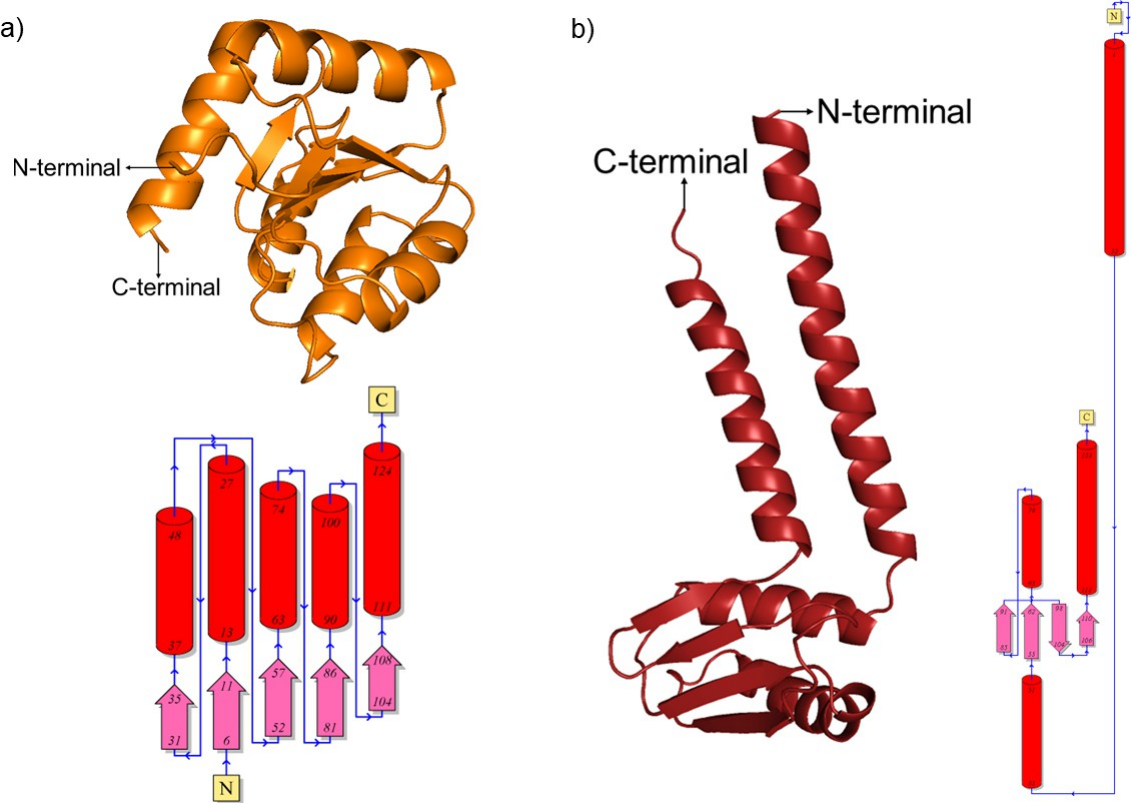
3D models and 2D topology diagrams of *Vibrio parahaemolyticus* proteins. (a) Chemotaxis CheY protein (Q79YX1), (b) HrpE/YscL family type III secretion apparatus protein (A0A6H0JL36). The red cylinder in the topology diagram represents α-helix, and the pink arrow indicates the β-strand to form β-sheet. A small blue arrow illustrates the direction of the protein chain from N- to C-terminal.

The 3D protein structures and 2D topology diagrams of ferritin, protein kinase domain-containing protein, and GPCR protein from *P*. *vannamei* (Fig 2) and HrpE/YscL family type III secretion apparatus protein, chemotaxis CheY protein from *V*. *parahaemolyticus* (Fig 3), which starts with N-terminal and end with C-terminal were retrieved from AlphaFold and PDBsum. For *P*. *vannamei* proteins, ferritin was found to be a single-domain structure with six α-helices (Fig 2a), protein kinase domain-containing protein is a single-domain structure with 19 α-helices and two β-sheets that contain seven β-strands (Fig 2b), and GPCR was determined to be a multi-domain structure with 24 α-helices and four β-sheets that comprise 18 β-strands (Fig 2c). Meanwhile, both *V*. *parahaemolyticus* proteins, chemotaxis CheY protein and HrpE/YscL family type III secretion apparatus, were identified as single-domain structures with five α-helices and one β-sheet containing five β-strands (Fig 3a), four α-helices and one β-sheet containing four β-strands, respectively (Fig 3b). The 3D protein structures of *P*. *vannamei* and *V*. *parahaemolyticus* retrieved from AlphaFold demonstrated that these proteins are reliable and reasonable as good protein structures, as they are located within a range of good protein structures according to several 3D structure validation tools, including ERRAT, PROCHECK, MolProbity, ProQ, and ProSA web server (S1 File).

### Protein complexes of *Penaeus vannamei*-*Vibrio parahaemolyticus*

The best-docked pose of protein complex for Complex 1 (Ferritin-HrpE/YscL family type III secretion apparatus protein) (S1 Fig), Complex 2 (Protein kinase domain-containing protein-Chemotaxis CheY protein) (S2 Fig), and Complex 3 (GPCR-Chemotaxis CheY protein) (S3 Fig) were predicted by HawkDock server with a docking score of -4319.76, -5271.39, and -4725.57, respectively (S1 Table).

H-bond, salt bridge (Table 1), and non-bonded contact interaction (S2 Table) with a distance within 5Å between *P*. *vannamei* and *V*. *parahaemolyticus* proteins residues were obtained using PyMOL tools and PDBsum. In Complex 1, the H-bond was formed between VAL154-SER42 and ARG153-VAL27 (S1a Fig), and 109 non-bonded contacts were formed in 24 interacting residues (S1b Fig). In Complex 2, H-bond was formed between THR20-HIS79, SER23-ASP51, GLY25-MET1, GLY25-ASN2, and PRO94-LYS78 (S2a Fig). Meanwhile, the salt bridge formation was identified between ASP100-ARG72, and 88 non-bonded contacts were formed between 24 interacting residues (S2b Fig). In complex 3, H-bond was formed between ASN35-SER14, ARG38-ASP12, ARG48-GLU34, and GLN368-ASP74 (S3a Fig), salt bridge bonds were identified between ARG38-ASP36, ARG41-ASP36, ARG48-GLU34, and 139 non-bonded contacts were formed in 29 interacting residues (S3b Fig).

**Table 1:** Distance of a hydrogen bond and a salt bridge between the interacting residues.

Protein complex	*Penaeus vannamei* residue	*Vibrio parahaemolyticus* residue	Interaction bond	Distance (Å)
Complex 1	VAL154	SER42	Hydrogen	2.60
	ARG153	VAL27	Hydrogen	2.60
Complex 2	THR20	HIS79	Hydrogen	2.99
	SER23	ASP51	Hydrogen	2.83
	GLY25	MET1	Hydrogen	2.74
	GLY25	ASN2	Hydrogen	2.74
	PRO94	LYS78	Hydrogen	2.95
	ASP100	ARG72	Salt bridge	3.81
Complex 3	ASN35	SER14	Hydrogen	2.89
	ARG38	ASP12	Hydrogen	3.23
	ARG48	GLU34	Hydrogen	3.14
	GLN368	ASP74	Hydrogen	3.14
	ARG38	ASP36	Salt bridge	3.64
	ARG41	ASP36	Salt bridge	3.64
	ARG48	GLU34	Salt bridge	3.14

### Molecular dynamics (MD) simulation of protein complexes

**Fig 4:**
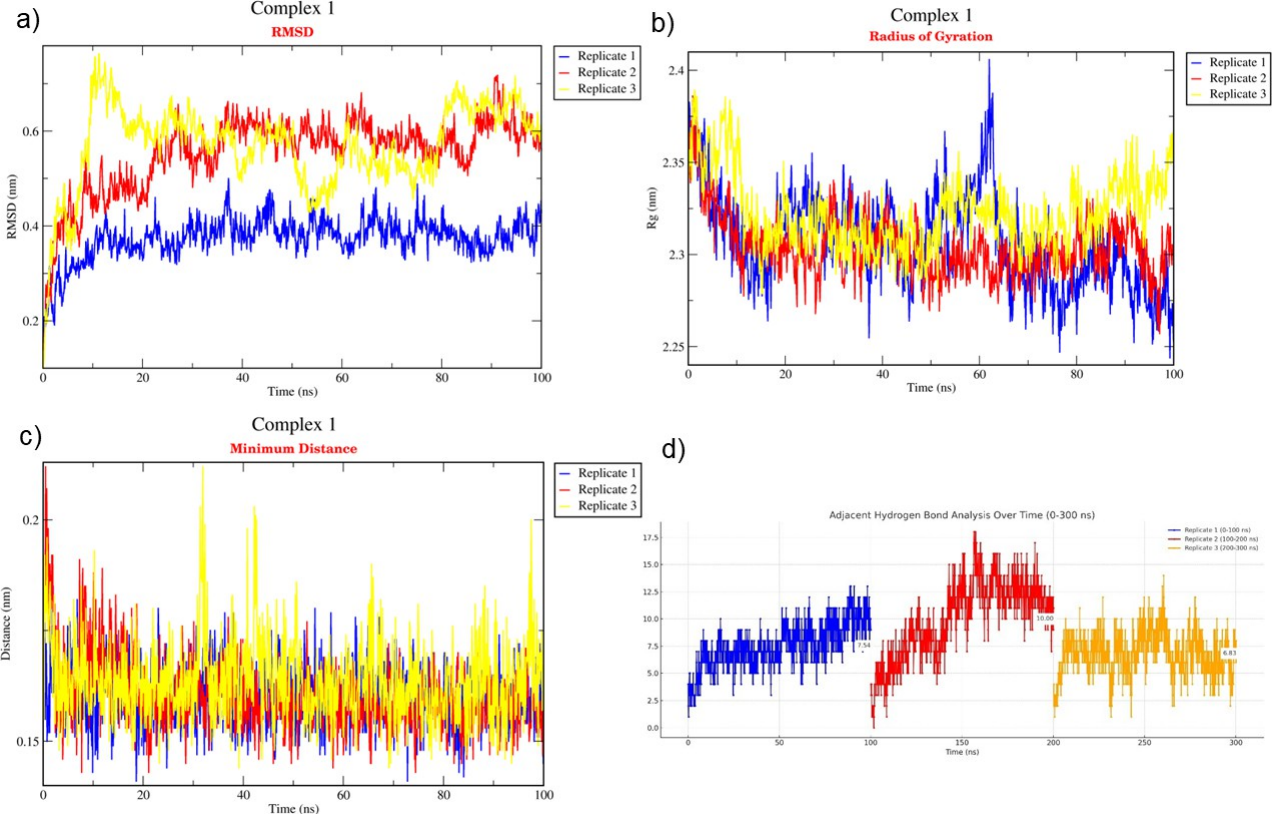
Triplicate of 100 ns MD simulation of Complex 1. (a) RMSD, (b) R_g_, (c) Distance, (d) H-bond. The blue plot indicates replicate 1, the red plot represents replicate 2, and the yellow plot illustrates replicate 3.

**Fig 5:**
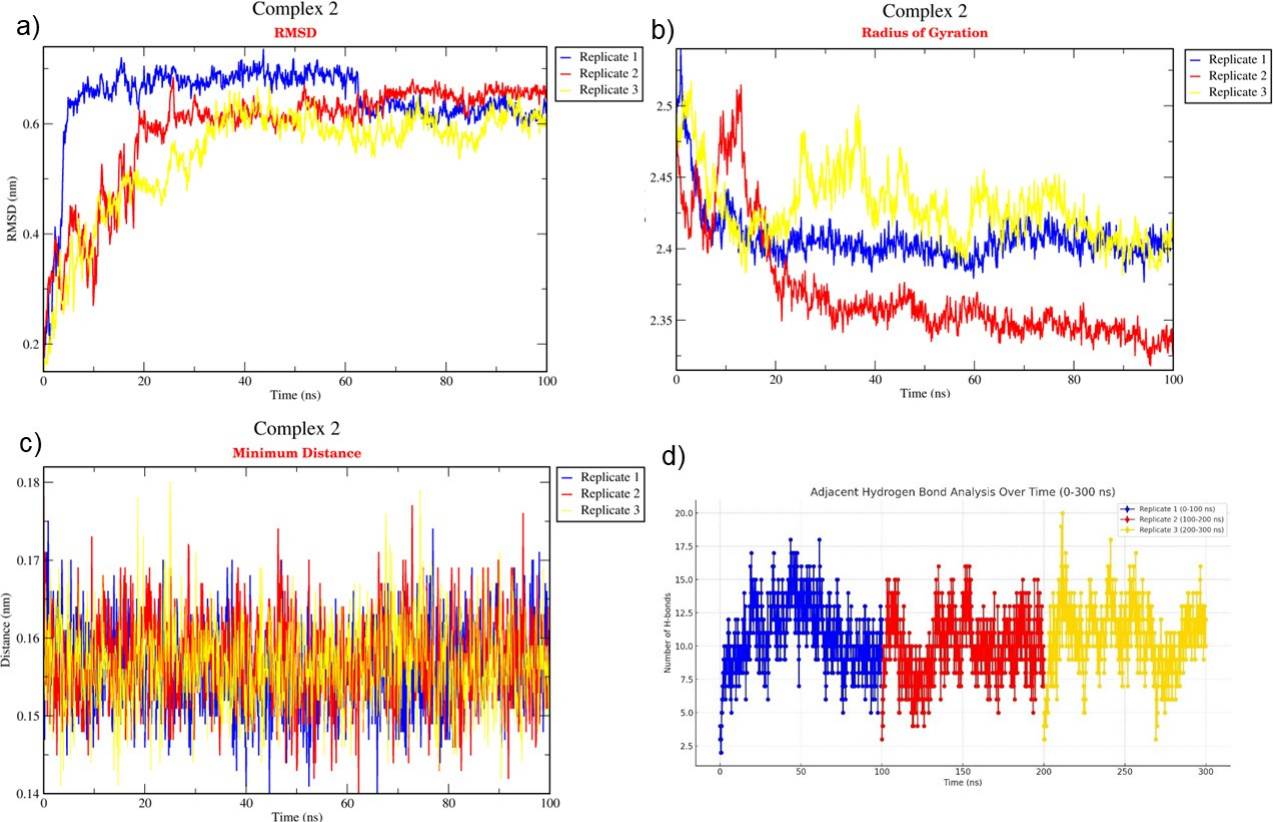
Triplicate of 100 ns MD simulation of Complex 2. (a) RMSD, (b) R_g_, (c) Distance, (d) H-bond. The blue plot indicates replicate 1, the red plot represents replicate 2, and the yellow plot illustrates replicate 3.

**Fig 6:**
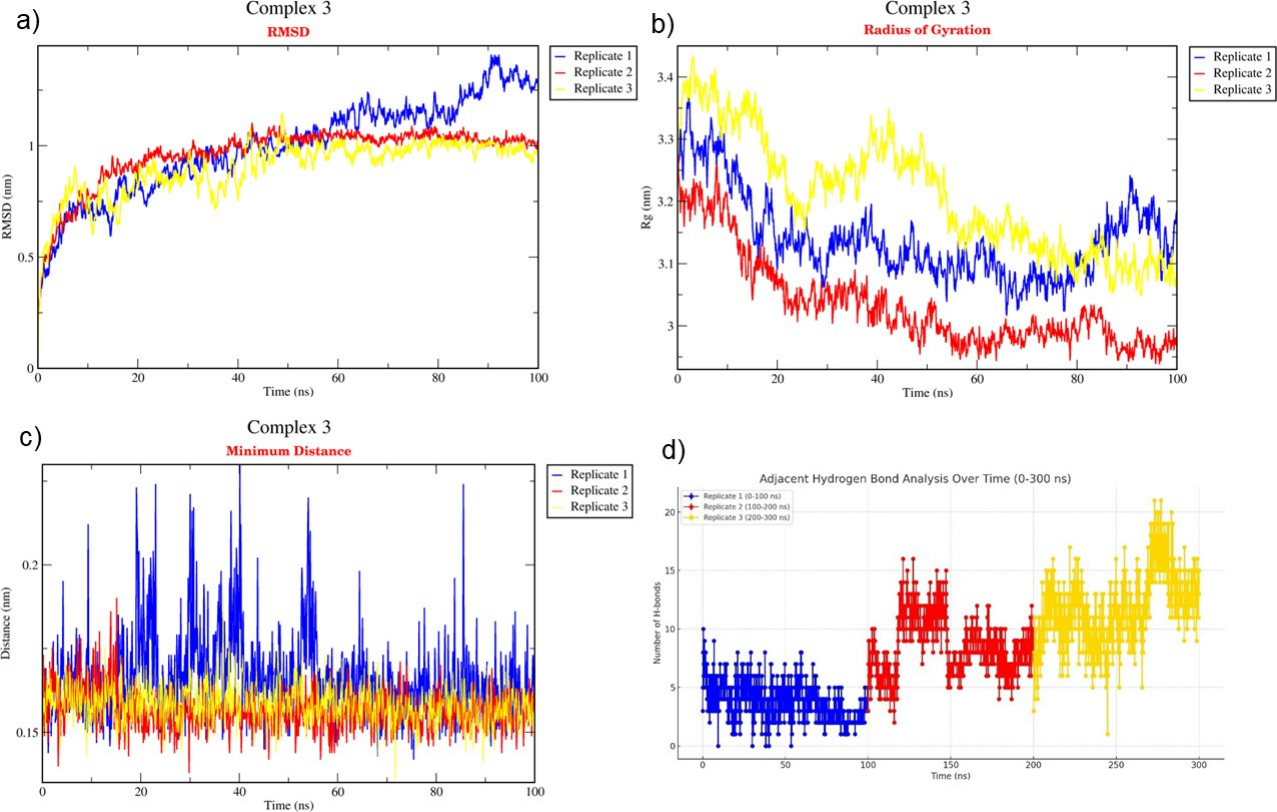
Triplicate of 100 ns MD simulation of Complex 3. (a) RMSD, (b) R_g_, (c) Distance, (d) H-bond. The blue plot indicates replicate 1, the red plot represents replicate 2, and the yellow plot illustrates replicate 3.

**Fig 7:**
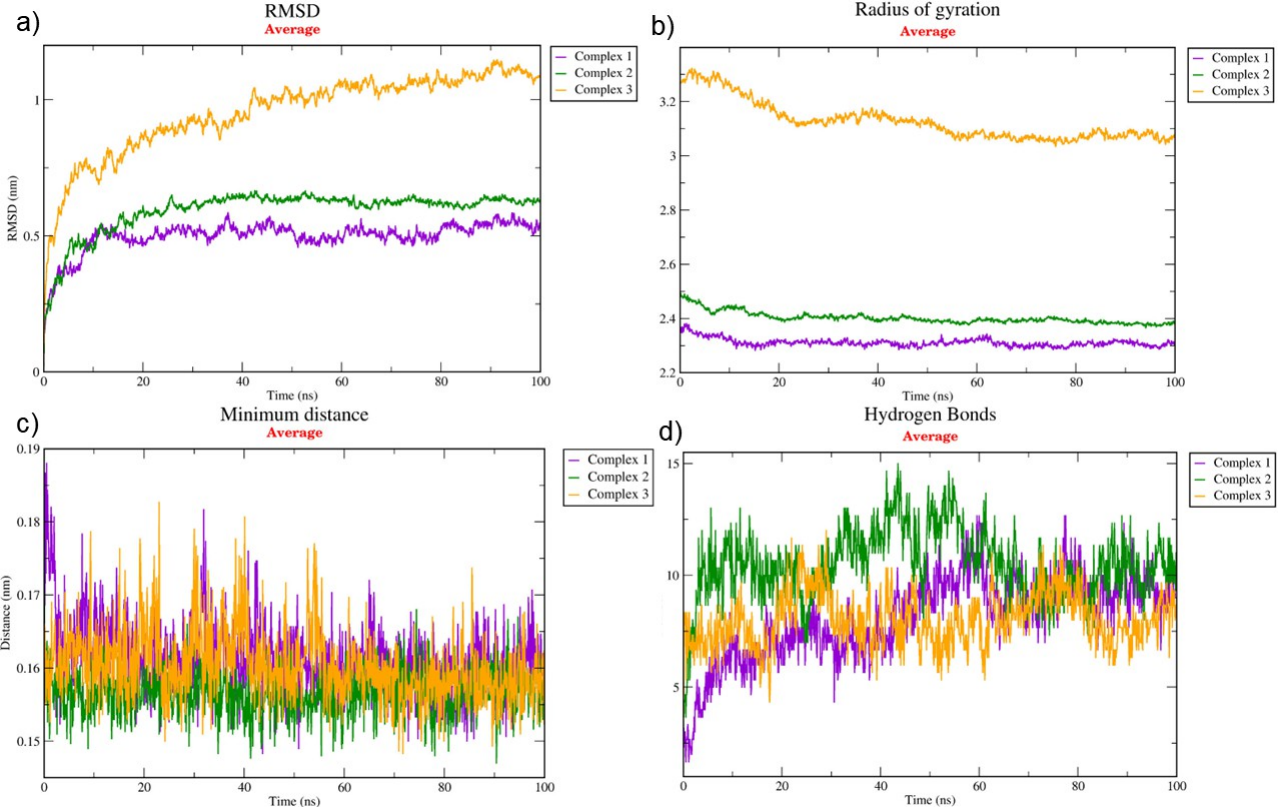
Average of triplicate 100 ns MD simulation. (a) RMSD, (b) R_g_, (c) H-bond, and (d) distance. The purple plot indicates Complex 1 (Ferritin-HrpE/YscL family type III secretion apparatus protein), the red plot represents Complex 2 (Protein kinase domain-containing protein-Chemotaxis CheY protein), and the orange plot illustrates Complex 3 (GPCR-Chemotaxis CheY protein).

**Fig 8:**
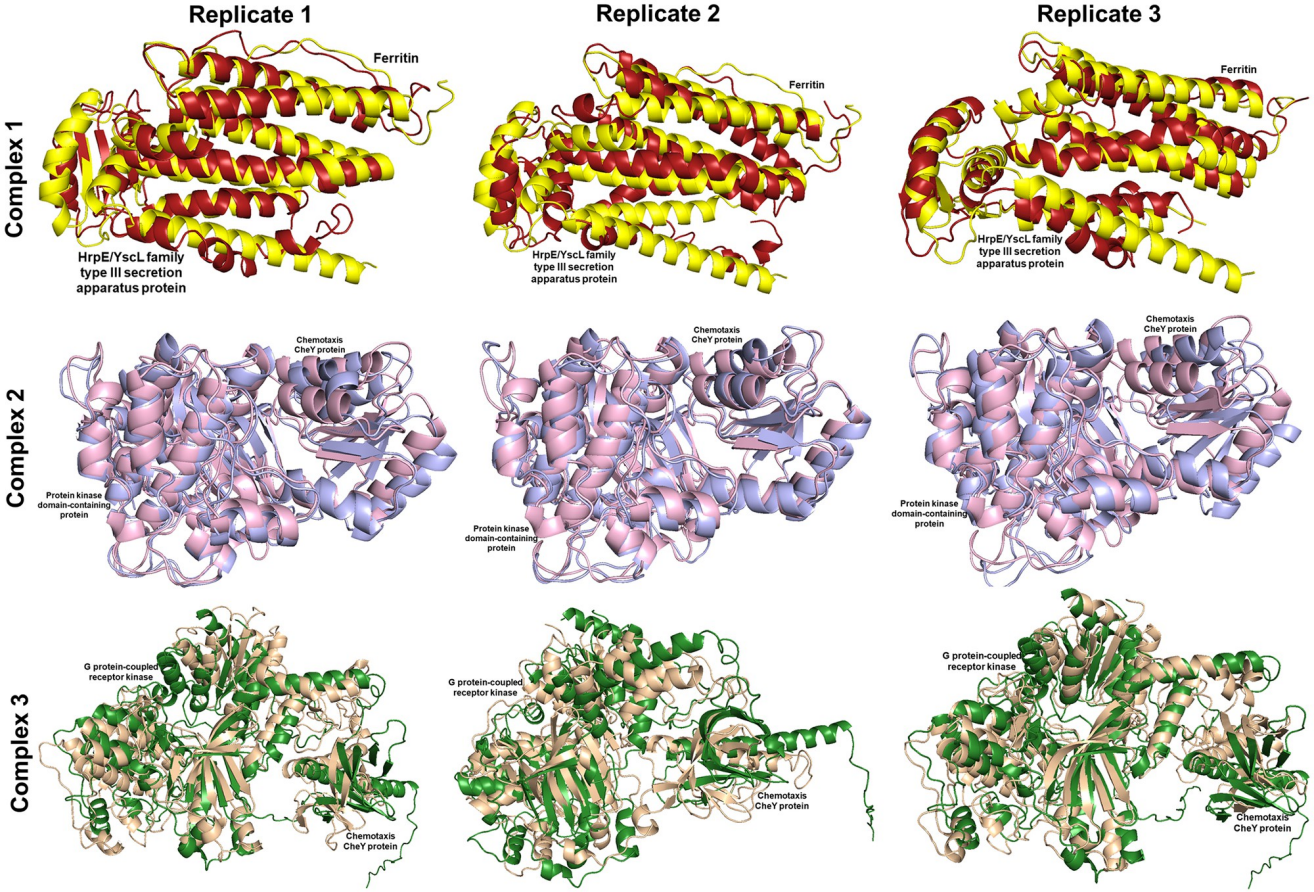
Superimposition of pre-MD and post-MD. The pre-MD for Complex 1, Complex 2, and Complex 3 are represented with yellow, pink, and green, respectively. Meanwhile, the post-MD for Complex 1, Complex 2, and Complex 3, are illustrated with red, purple, and brown, respectively.

The binding affinity of each protein complex of *P*. *vannamei* and *V*. *parahaemolyticus* was determined using triplicate MD simulation over 100 ns (Figs 4–6) by analysing the average RMSD, H-bond, R_g_, and distance. During the 100 ns simulation, RMSD values of Complex 1, Complex 2, and Complex 3 were 0.530 nm, 0.632 nm, and 1.088 nm, respectively (Fig 7a). The rigidity of each complex was analysed based on the R_g_ with a value of 2.302 nm, 2.381 nm, and 3.076 nm, accordingly (Fig 7b). Nine H-bonds were identified in Complex 1 and Complex 2, and eight H-bonds were identified in Complex 3 (Fig 7c) during 100 ns simulation, where the significance of interaction refers to high H-bond formation. After 100 ns simulation, the distance between *P*. *vannamei* and *V*. *parahaemolyticus* proteins in Complex 1 and Complex 3 was 0.150 nm, while in Complex 2 the distance between both proteins was 0.153 nm (Fig 7d). After 100 ns MD simulation, each complex undergoes conformational changes in each replicate, which is represented in Fig 8.

### Principal component analysis (PCA) of molecular dynamics (MD) simulation trajectories

**Fig 9:**
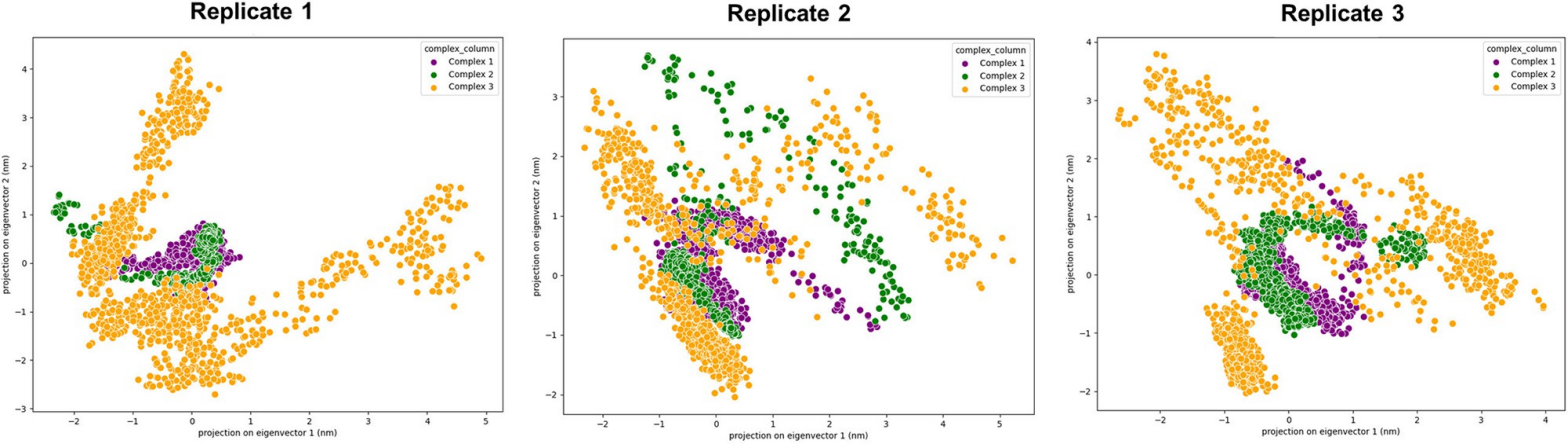
Scatter plot of PCA over 100 ns MD simulation. The purple plot represents Complex 1, the green plot indicates Complex 2, and the yellow plot shows Complex 3.

The scatter plot represented in Fig 9 visualises the projection of the MD simulation trajectory onto the first two principal components, PC1 and PC2, which showed a significantly large motion of Complex 3 compared with Complex 1 and Complex 2. Referring to Fig 9, each data point in the plot represents a distinct conformation of the protein complex at a specific time point in the simulation, mapped in terms of its deviation along the major axes of conformational variability. The PC1 axis (horizontal) captures the most significant mode of structural variation, encompassing large-scale conformational changes, while the PC2 axis (vertical) represents the second most dominant mode, often associated with more subtle or local fluctuations. The distribution and density of the points across this 2D space provide a visual representation of the conformational diversity explored by the protein, highlighting regions of conformational stability (dense clusters) and transition (sparse or bridge-like connections between clusters).

### Clustering analysis of molecular dynamics (MD simulation trajectories)

**Fig 10:**
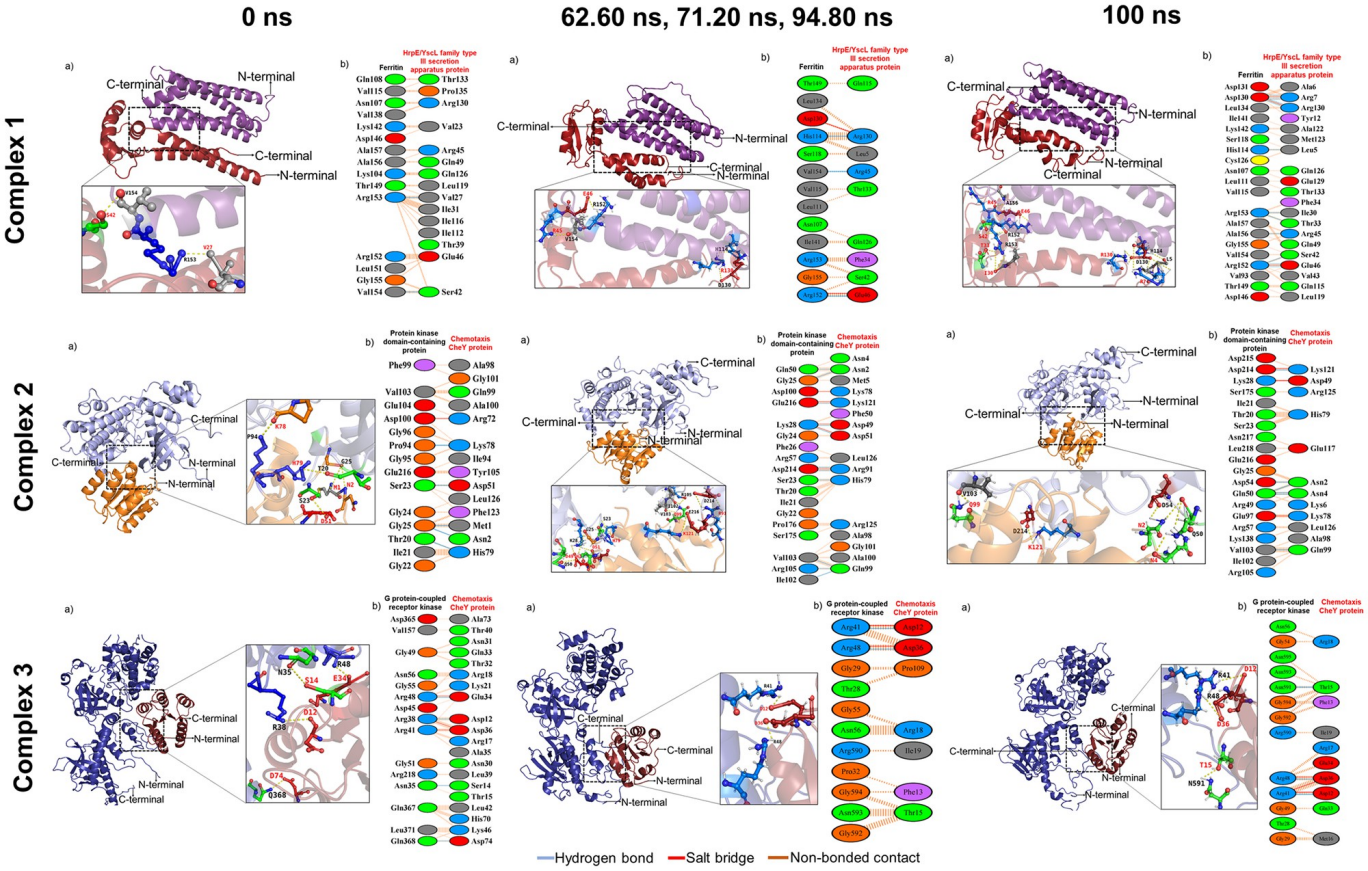
Comparative PPI of the top cluster coordinates from MD simulation trajectories.

The clustering analysis of Complex 1 formed 11 clusters with an average RMSD of 0.251 nm, Complex 2 formed 8 clusters with a 0.229 nm average RMSD, and 73 clusters with a 0.560 nm average RMSD formed in Complex 3. From the clusters, the most dominant structure of Complex 1, Complex 2, and Complex 3, located in time frames of 62.60 ns, 71.20 ns, and 94.80 ns, respectively, throughout the 100 ns simulation, which these dominant structure were located in replicate 1 in each complex. The comparative interaction of different cluster coordinates, Complex 1 (0 ns, 62.60 ns, 100 ns), Complex 2 (0 ns, 71.20 ns, 100 ns), and Complex 3 (0 ns, 94.80 ns, 100 ns), showed the changes of protein conformational and the total of *P*. *vannamei*-*V*. *parahaemolyticus* PPI (Fig 10).

### Molecular mechanics poisson-boltzmann surface area (MM/PBSA) calculation

MM/PBSA calculated the binding free energy in VDWAALS, EEL, EGB, and ESURF (Table 2) after each complex (Complex 1, Complex 2, Complex 3) was stabilised *via* simulation. The average total binding energy of Complex 1, Complex 2, and Complex 3 is -22.50 kJ/mol, -30.20 kJ/mol, and -26.27 kJ/mol, respectively.

**Table 2:** Average MM/PBSA free energy of *Penaeus vannamei-Vibrio parahaemolyticus* complex of triplicate MD simulation.

Complex	VDWAALS (kJ/mol)	EEL (kJ/mol)	EGB (kJ/mol)	ESURF (kJ/mol)	GGAS (kJ/mol)	GSOLV (kJ/mol)	Average (kJ/mol)
Complex 1	-65.47	115.71	-64.32	-8.41	50.24	-72.74	-22.50
Complex 2	-89.30	-395.90	467.52	-12.52	-485.21	455.00	-30.20
Complex 3	-48.81	-239.48	271.10	-9.07	-288.30	262.02	-26.27

## Discussion

In-depth knowledge of the interactions between host and pathogen proteins is essential to uncover the mechanisms of bacterial infection that may contribute to the advancement of shrimp farming production [54]. The information on the PPI between proteins in *P*. *vannamei* and *V*. *parahaemolyticus* is able to deepen the understanding of how *V*. *parahaemolyticus* interacts, evades, and invades the host’s immune system. This knowledge may be essential in the development of treatment and prevention strategies to fight pathogenic infection in shrimp farming, as this problem remains a major challenge to the success of shrimp aquaculture production [55]. In this study, the interactions between *P*. *vannamei* and *V*. *parahaemolyticus* proteins, i.e., ferritin-HrpE/YscL family type III secretion apparatus protein, protein kinase domain-containing protein-chemotaxis CheY protein, GPCR-chemotaxis CheY protein were elucidated using a structure-based approach by identifying the binding region that involved several amino acid residues, which suggest these interacting residues might taking part in infection.

Ferritin, a major storage protein for iron, is crucial in energy metabolism, nucleic acid synthesis, and cell proliferation in all organisms [56]. This protein can also protect cells from the detrimental effects of free iron, where the availability of free iron promotes cell and tissue damage *via* free radical formation [57]. During an infection, both host and pathogen compete for available nutrients, such as iron, which is stored within the ferritin protein. Thus, in response to infection, the host protects its iron metabolism by limiting its availability to the bacteria to prevent bacteria replication [58]. However, in several bacteria species, including *V*. *parahaemolyticus*, *Yersinia pestis*, and *Salmonella enterica serovar* Typhimurium, ferritin is used as an iron source during infection and damages the host’s membrane by bacterial secretion system, T3SS [59].

Meanwhile, protein kinases are vital in crustacean cellular processes, such as cell division, proliferation, apoptosis, and differentiation, especially during immune response [60]. Protein kinases are also involved in the regulation of flagella length during the initiation of infection in the host [61, 62]. In bacterial infection, kinase proteins, known as mitogen-activated protein kinases, suppress the inflammatory reactions in orange-spotter grouper, *Epinephelus coioides*, which were infected with the flagellar basal-body rod protein (*flgC*) gene associated with chemotaxis and motility regulation [63]. In addition, CheY is a gene of chemotaxis that acts as a response regulator, which controls the motility of bacterial flagellar through the binding process to the cytoplasmic switch complex of the flagellar motor [64].

GPCR is vital in crustacean immune processes and aids in host defence, as indicated by its up-regulation after being challenged by gram-positive bacteria [65]. A GPCR family member, CXCR2, was also found to bind with the interleukin-8 (IL8) receptor during *V*. *parahaemolyticus* infection in large yellow croaker, *Larimichthys crocea*, in which the interaction initiated both chemotaxis and phagocytosis processes [66]. The involvement of these proteins during infection suggests their interactions might mediate the cross-talks between *P*. *vannamei* and *V*. *parahaemolyticus*. Hence, the interactions between these proteins were structurally observed to determine their specific binding sites and affinity.

Protein-protein docking is essential in the identification of possible binding sites of protein pairs [67]. Using the validated retrieved 3D structures from AlphaFold, the interactions between three protein pairs in *P*. *vannamei* and *V*. *parahaemolyticus*, namely Complex 1 (Ferritin-HrpE/YscL family type III secretion apparatus protein), Complex 2 (Protein kinase domain-containing protein-Chemotaxis CheY protein), and Complex 3 (GPCR-Chemotaxis CheY protein), were docked to predict their binding site and the best-docked of complexes were ranks according to docking score. Docked complexes were ranked based on the top 10 docked scores, which is important to validate the binding mode and to predict the binding affinity [68], and the docked pose with the lowest score in the top 10 ranks indicates the best-docked complexes [42]. The specific binding sites identified in the *P*. *vannamei-V*. *parahaemolyticus* protein complexes with a distance of interacting residues within 5Å are defined as closely interacting [69]. The *P*. *vannamei* and *V*. *parahaemolyticus* complexes that consist of Complex 1, Complex 2, and Complex 3 indicate that the H-bond is formed in all complexes between specific interacting residues. H-bond is pivotal in controlling molecular interaction and is significant in protein-protein binding, thereby determining the specificity between *P*. *vannamei* and *V*. *parahaemolyticus* [70, 71]. The identified H-bond in the host-pathogen interaction involving specific residues indicates strong binding due to the higher binding affinity, suggesting the binding is important for the infection of pathogenic agents into the host cells. This mechanism is illustrated in the respiratory syncytial virus (RSV) and the severe acute respiratory syndrome coronavirus (SARS-CoV) infection in humans [72, 73], and the WSSV infection in crustaceans [25, 74].

Other types of intermolecular bonds in PPI, such as salt bridges and non-bonded contacts, are also important, and the information can be obtained in PDBSum. These bonds are important to maintain the structure’s stability and rigidity [71, 75–77]. Salt bridges are also highly involved in protein folding, as highlighted in host-pathogen studies investigating the innate immune response and adaptation of an antarctic notothenioid fish, *Trematomus bernacchii*, during bacterial infection in low-temperature environments [78]. Non-bonded contacts contribute to the stabilisation of insulin-like growth factor binding proteins (IGFBPs) and insulin-like growth factor (IGF) binding, suggesting the specific interaction interface that mediates the understanding of insulin endocrinology in the eastern spiny lobster, *Sagmariasus verreauxi* [79]. These examples show the importance of the salt bridges and non-bonded contact interactions in understanding how proteins interact with functional residues that are involved in the stabilisation of the protein complex and binding to the specific residues in protein complexes [71].

The results from this study identified significant interacting residues in specific binding sites and bond formation that occur between selected proteins of *P*. *vannamei* and *V*. *parahaemolyticus*, which can be experimentally validated to measure their strength and involvement in executing their functions. However, the interaction of H-bond is significantly relevant in drug discovery and development in future studies, which affects the biological activity, pharmacokinetics, and physicochemical properties of drugs [80] that are able to inhibit the pathogen infection compared to the salt bridge and non-bonded interactions as their roles in drug design have remained unclear [76]. In current study, the interacting residues that involved H-bond formation in each complex, Complex 1 (VAL154-SER42, ARG153-VAL27), Complex 2 (THR20-HIS79, SER23-ASP51, GLY25-MET1, GLY25-ASN2, PRO94-LYS78), and Complex 3 (ASN35-SER14, ARG38-ASP12, ARG48-GLU34, GLN368-ASP74) might be a crucial region for inhibition of pathogenic infection. In addition, the MD simulation can further validate the binding affinity of *P*. *vannamei-V*. *parahaemolyticus* complexes that reveal the strength of their interaction by several parameters, including the H-bond.

The triplicate MD simulation with 100 ns trajectories generates RMSD, R_g_, H-bond, and distance that are used to measure the stability and behaviour of protein complexes. The RMSD values of Complex 1, Complex 2, and Complex 3 were 0.530 nm, 0.632 nm, and 1.088 nm, respectively. The RMSD graph (Fig 7a) shows a steady and constant plot without huge spikes, suggesting that the protein complex remained stable throughout the 100 ns simulation. Low RMSD values indicate that the protein complex is highly stable [81]. However, based on the results, Complex 3 has a significantly higher RSMD value than Complex 1 and Complex 2 due to the larger size of the protein complex, which could influence the higher RMSD [82]. In terms of molecular dynamics, a larger protein complex frequently exhibits more dynamic behaviour by involving conformational changes, protein movements, and interactions. The R_g_ is an important parameter that indicates the compactness and proper folding of the protein [83, 84]. In the present study, the R_g_ values of each protein complex were 2.250 nm, 2.398 nm, and 3.161 nm, which depicted the lower R_g_ values, proving the high stability of the complex and revealing the compactness is consistent during 100 ns simulation [84]. H-bond between selected proteins in *P*. *vannamei* and *V*. *parahaemolyticus* proteins are calculated to evaluate the binding affinity and confirm the stable interaction [85]. Nine H-bonds in Complex 1 and 2 and eight H-bonds in Complex 3 suggest strong binding stability [23]. The distances between selected proteins in *P*. *vannamei* and *V*. *parahaemolyticus* proteins are calculated to confirm their binding affinities. We calculated the distances between two proteins in each complex, i.e., 0.148 nm for Complex 1 and Complex 2 and 0.151 nm for Complex 3. A distance below 3.5Å reveals a strong binding affinity between proteins [86]. Based on the RMSD, R_g_, H-bond, and distance values, Complex 1, Complex 2, and Complex 3 are highly stable and exhibit strong binding with each other.

PCA is a widely used approach to determine the dominant molecular motion in the MD trajectory [87] and offers a compact yet insightful view into the dynamic behaviour of Complex 1, Complex 2, and Complex 3 during the simulation period performed in this study. Notably, several distinct clusters can be observed, indicative of preferred conformational states or energy wells where the protein spends a significant portion of its simulation time [88]. The central cluster, characterised by a high density of data points, likely represents the most energetically favourable and most frequently adopted conformation under the simulated conditions. Its centrality and density suggest a conformational state with considerable stability, potentially correlating with the protein’s functional resting or inactive state. The extent of spread along the PC1 axis underscores the protein’s ability to undergo large-scale conformational rearrangements, which could be critical for its biological function, such as ligand binding, allosteric regulation, or PPI [89]. In contrast, the spread along the PC2 axis, while covering a smaller range, indicates additional degrees of conformational freedom [89]. These might correspond to more localised movements, such as side-chain rearrangements, loop flexibilities, or domain swiveling, complementing the broader structural transitions captured by PC1. Furthermore, the presence of points forming less dense regions or paths between the main clusters is particularly intriguing. These points may represent transitional conformations that the protein adopts as it shifts from one stable state to another. Such transitions are often crucial in understanding the mechanism of action of the protein, as they can reveal the pathways through which conformational changes propagate [90]. Identifying these transitional states can provide key insights into the dynamic aspects of the protein’s function, offering potential targets for therapeutic intervention or modulation.

In addition, the MD simulation generates millions of data, making it difficult to determine each complex’s most representative protein conformations. The clustering analysis is one of the advanced analyses used to identify the most dominant protein conformation throughout the simulation in the specific time frames, which is pivotal in elucidating the behaviour of protein complexes [91]. The comparative interaction across trajectories demonstrated changes in the interaction between *P*. *vannamei* and *V*. *parahaemolyticus* proteins as a result of protein complex conformational changes throughout the simulation, which could aid in a better understanding of the interactions. The MM-PBSA analysis is an end-point for docking pose evaluation, structure stability, and binding affinity determination by calculating the binding free energy in VDWAALS, EEL, EGB, and ESURF between the selected proteins in *P*. *vannamei* and *V*. *parahaemolyticus* [52]. In this study, VDWAALS, EEL, and ESURF contribute negatively and favour protein binding in Complex 2 and Complex 3, while the contribution of EGB was very low. In complex 1, the protein binding was negatively contributed by VDWAALS, EGB, and ESURF, while there are comparatively low EEL contributions. The results indicate the high binding affinity was -30.20 kJ/mol in Complex 2, followed by Complex 3 and Complex 1 with the average binding energy of -26.27 kJ/mol and -22.50 kJ/mol, respectively. It suggests the high binding stability of the interaction between *P*. *vannamei* and *V*. *parahaemolyticus* due to the contribution of VDWAALS, EEL, EGB, and ESURF.

## Conclusion

Protein-protein docking and MD simulation might provide the structural insight into host-pathogen interactions between *P*. *vannamei* and *V*. *parahaemolyticus*. The protein-protein docking predicts the structure of protein complexes and identifies the several interacting residues, specifically, the binding sites formed by H-bond during bacterial infection consist of VAL154-SER42, ARG153-VAL27 in Complex 1, THR20-HIS79, SER23-ASP51, GLY25-MET1, GLY25-ASN2, PRO94-LYS78 in Complex 2, and ASN35-SER14, ARG38-ASP12, ARG48-GLU34, GLN368-ASP74 in Complex 3 that might be a target region for drug development in the future study. From this study, protein binding in each complex is stable and strong, based on their RMSD, R_g_, H-bond, and distance value during 100 ns MD simulation and MM-PBSA analysis used as an end-point in validating binding affinity revealed that the interaction of Complex 1, Complex 2, and Complex 3 was highly stable and strongly bind. Hence, this suggests that significant binding causes infection in *P*. *vannamei*. In-depth knowledge of the molecular interactions between the host and pathogen has improved our understanding of the mechanisms of bacterial infection, and it can be used in the biomarker identification in shrimp diseases and to develop strategies for effective treatment by targeting specific binding sites involved in the host-pathogen interactions.

## Supporting information

S1 Fig Complex 1 (ferritin-HrpE/YscL family type III secretion apparatus protein). The purple chain indicates the ferritin protein from *P*. *vannamei*, and the red chain indicates the HrpE/YscL family type III secretion apparatus protein from *V*. *parahaemolyticus*.
(TIF)

S2 Fig Complex 2 (protein kinase domain-containing protein-chemotaxis CheY protein). The blue chain indicates the protein kinase domain-containing protein from *P*. *vannamei*, and the maroon chain indicates the chemotaxis CheY protein from *V*. *parahaemolyticus*.
(TIF)

S3 Fig Complex 3 (GPCR-chemotaxis CheY protein). The purple chain indicates the GPCR protein from *P*. *vannamei*, and the orange chain indicates the chemotaxis CheY protein from *V*. *parahaemolyticus*.
(TIF)

S1 File Protein structure validation using ERRAT, PROCHECK, MolProbity, ProQ, and ProSA. (DOCX)

S1 Table Protein-protein docking score using the HawkDock server. (DOCX)

S2 Table Distance of non-bonded contact between *Penaeus vannamei* and *V*. *parahaemolyticus* residues. (DOCX)
