# Supplementary figures and images for: Molecular docking and dynamics simulation studies uncover the host-pathogen protein-protein interactions in Penaeus vannamei and Vibrio parahaemolyticus
Source: PLoS One. 2024 Jan 24;19(1):e0297759. doi: 10.1371/journal.pone.0297759 (PMC10807825; doi:10.1371/journal.pone.0297759)

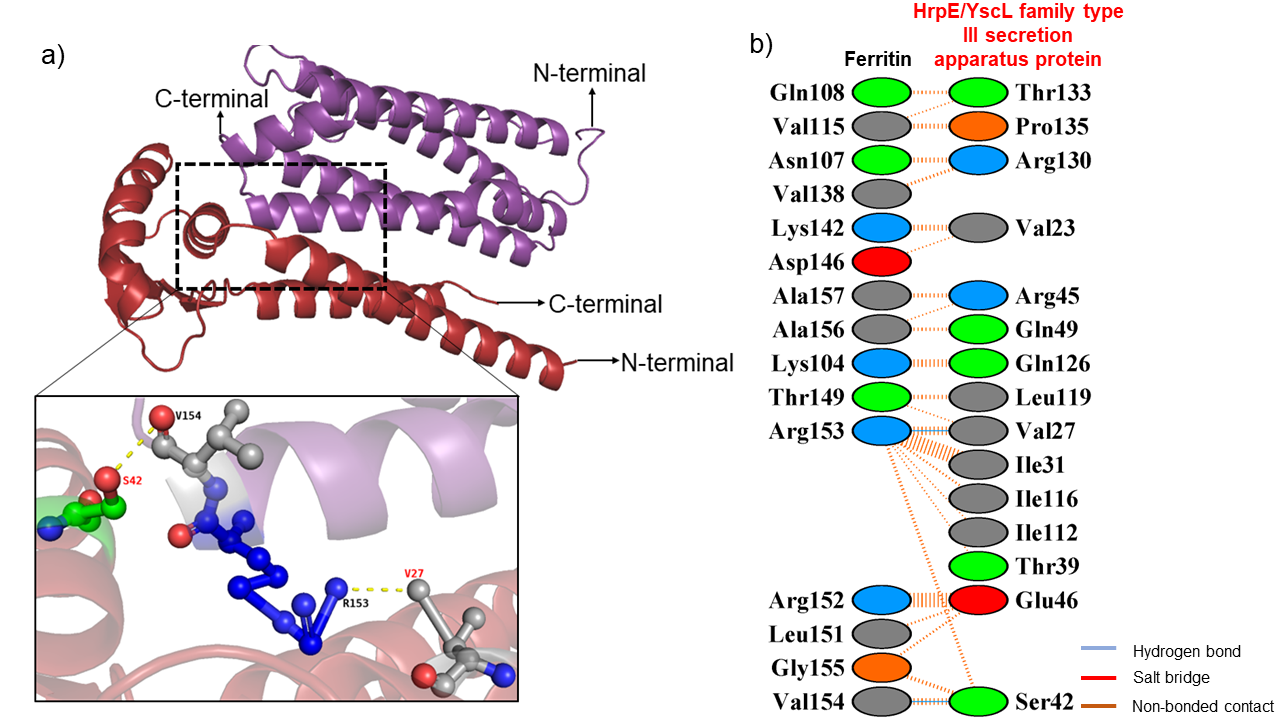

Supplement: S1 Fig — The purple chain indicates the ferritin protein from P. vannamei, and the red chain indicates the HrpE/YscL family type III secretion apparatus protein from V. parahaemolyticus. (TIF) [file pone.0297759.s001.tif]

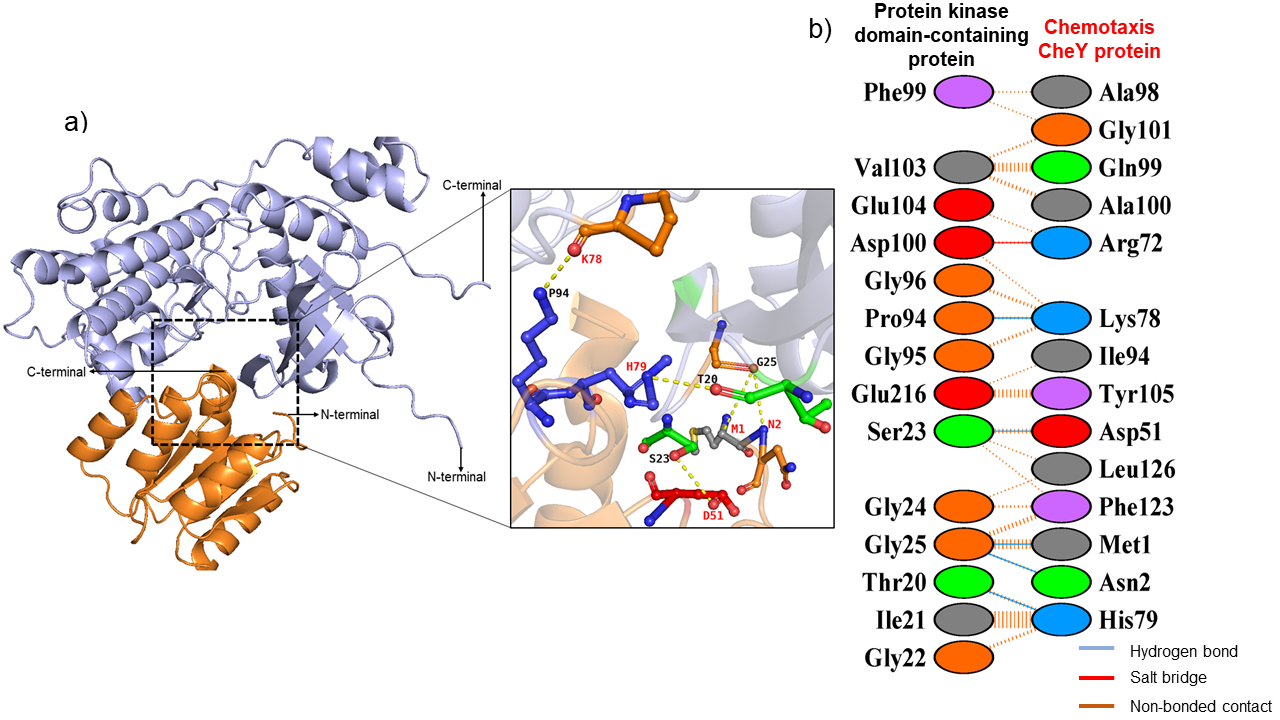

Supplement: S2 Fig — The blue chain indicates the protein kinase domain-containing protein from P. vannamei, and the maroon chain indicates the chemotaxis CheY protein from V. parahaemolyticus. (TIF) [file pone.0297759.s002.tif]

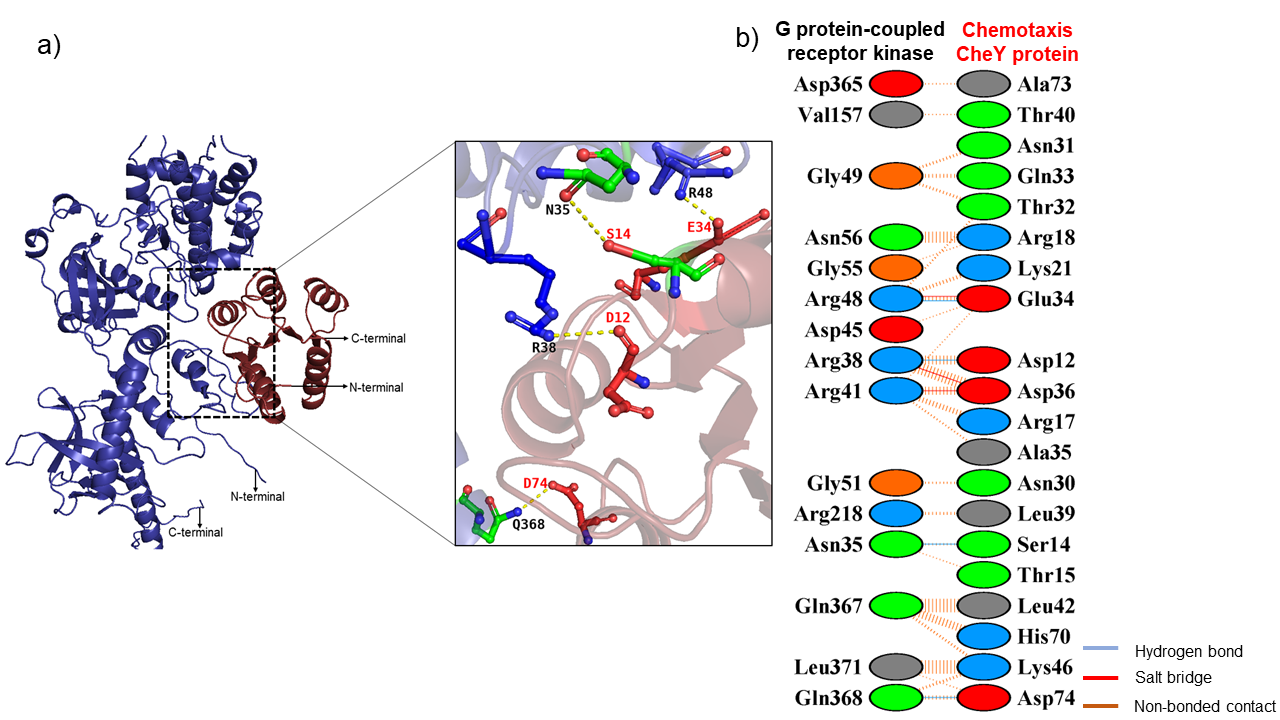

Supplement: S3 Fig — The purple chain indicates the GPCR protein from P. vannamei, and the orange chain indicates the chemotaxis CheY protein from V. parahaemolyticus. (TIF) [file pone.0297759.s003.tif]
