# Supplementary material for: Molecular docking and dynamics simulation studies uncover the host-pathogen protein-protein interactions in Penaeus vannamei and Vibrio parahaemolyticus
Source: PLoS One. 2024 Jan 24;19(1):e0297759. doi: 10.1371/journal.pone.0297759 (PMC10807825; doi:10.1371/journal.pone.0297759)
Supplement: S1 File — (DOCX) [file pone.0297759.s004.docx]

**Table 1.** **Protein structure validation using ERRAT, PROCHECK, MolProbity, ProQ, and ProSA.**

| Protein structure | ERRAT (%) | PROCHECK (%) | MolProbity  (Å) | ProQ  (LGscore) | ProSA  (z-score) |
| --- | --- | --- | --- | --- | --- |
| Ferritin | 93.79 | 94.7 | 0.74  (100^th^ percentile) | 10.47 | -6.73 |
| Protein kinase domain-containing protein | 98.76 | 90.0 | 0.96  (100^th^ percentile) | 9.49 | -8.18 |
| GPCR | 90.39 | 90.3 | 1.39  (97^th^ percentile) | 9.58 | -10.92 |
| HrpE/YscL family type III secretion apparatus protein | 100 | 96.8 | 1.01  (100^th^ percentile) | 11.83 | -5.73 |
| Chemotaxis CheY protein | 95.76 | 95.6 | 1.17  (99^th^ percentile) | 5.86 | -5.87 |


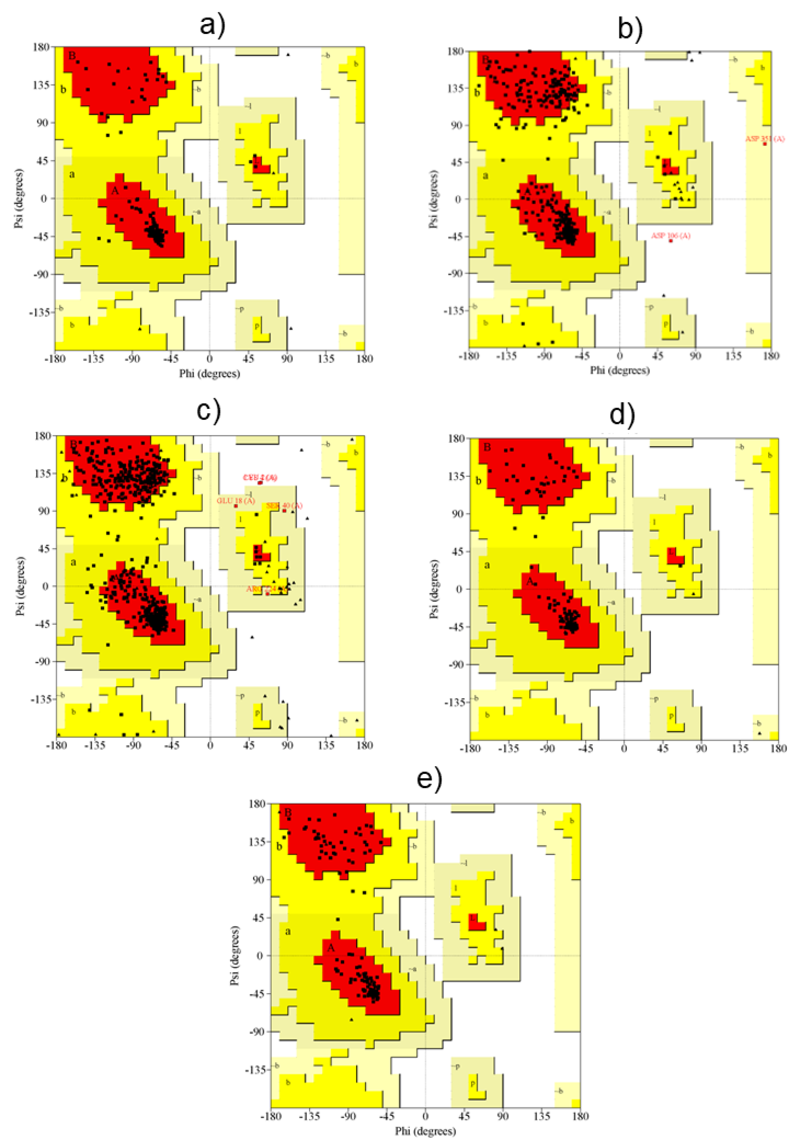
**Fig 1**. **Ramachandran plots generated by PROCHECK.** (a) Ferritin, (b) Protein kinase domain-containing protein, (c) GPCR, (d) HrpE/YscL family type III secretion apparatus protein, and (e) Chemotaxis CheY protein. The red regions indicate the most favoured region, the yellow regions represent additional allowed regions, the pale-yellow regions show generously allowed regions, and the white regions indicate disallowed regions.
