# Supplementary material for: Molecular docking and dynamics simulation studies uncover the host-pathogen protein-protein interactions in Penaeus vannamei and Vibrio parahaemolyticus
Source: PLoS One. 2024 Jan 24;19(1):e0297759. doi: 10.1371/journal.pone.0297759 (PMC10807825; doi:10.1371/journal.pone.0297759)
Supplement: S1 Table — (DOCX) [file pone.0297759.s005.docx]

**S1 Table**. **Protein-protein docking score using the HawkDock server.**

| Protein complex  (*Penaeus vannamei-Vibrio parahaemolyticus*) | Docking score |
| --- | --- |
| Complex 1  (Ferritin- HrpE/YscL family type III secretion apparatus protein) | -4319.76 |
| Complex 2  (Protein kinase domain-containing protein-Chemotaxis CheY protein) | -5271.39 |
| Complex 3  (GPCR-Chemotaxis CheY protein) | -4725.57 |
