# Supplementary material for: Molecular docking and dynamics simulation studies uncover the host-pathogen protein-protein interactions in Penaeus vannamei and Vibrio parahaemolyticus
Source: PLoS One. 2024 Jan 24;19(1):e0297759. doi: 10.1371/journal.pone.0297759 (PMC10807825; doi:10.1371/journal.pone.0297759)
Supplement: S2 Table — (DOCX) [file pone.0297759.s006.docx]

**S2 Table. Distance of non-bonded contact between Penaeus vannamei and V. parahaemolyticus residues.**

| Protein complex | *Penaeus vannamei* residue | *Vibrio parahaemolyticus* residue | Distance (Å) |
| --- | --- | --- | --- |
| Complex 1 | LYS104 | GLN126 | 3.57, 3.75, 3.15, 3.87, 3.39, 2.75, 3.51, 3.36 |
|  | ASN107 | ARG130 | 3.66, 3.69, 3.74, 3.72, 2.86 |
|  | GLN108 | THR133 | 3.89, 3.79, 3.41 |
|  | VAL115 | PRO135 | 3.67, 2.78, 3.65, 3.31, 3.04 |
|  | VAL115 | THR133 | 3.73 |
|  | VAL138 | ARG130 | 3.22, 2.93, 2.66 |
|  | LYS142 | VAL23 | 3.17, 2.69, 2.54, 3.34, 2.43, 3.03 |
|  | ASP146 | VAL23 | 3.61 |
|  | THR149 | LEU119 | 3.84, 3.48, 3.13, 3.79 |
|  | THR149 | VAL27 | 3.66 |
|  | LEU151 | GLU46 | 3.48, 2.99 |
|  | ARG152 | GLU46 | 3.23, 3.04, 3.12, 2.28, 3.19, 2.67, 3.73, 2.56, 2.53, 2.46, 3.62, 3.19, 3.28, 3.64, 3.69 |
|  | ARG153 | SER42 | 3.56, 2.70, 3.39 |
|  | ARG153 | THR39 | 3.88 |
|  | ARG153 | ILE31 | 3.75, 3.87, 2.59, 2.57, 3.75, 2.41, 1.56, 3.35, 2.00, 1.06, 3.46, 3.59, 2.86, 1.51, 1.73, 3.02, 1.76 |
|  | ARG153 | ILE112 | 3.80 |
|  | ARG153 | VAL27 | 3.52, 2.89, 3.36, 2.60, 3.03, 3.63, 3.56, 3.62, 2.30 |
|  | ARG153 | ILE116 | 3.41, 3.54 |
|  | VAL154 | SER42 | 3.90, 3.48, 3.44, 2.97, 3.04, 3.07, 2.69 |
|  | GLY155 | SER42 | 3.25, 3.81, 3.47, 3.75 |
|  | GLY155 | GLU46 | 3.87, 3.77 |
|  | ALA156 | ARG45 | 3.71 |
|  | ALA156 | GLN49 | 3.45, 3.33, 2.76 |
|  | ALA157 | ARG45 | 3.74, 3.86, 3.66, 3.89 |
|  |  |  |  |
| Complex 2 | THR20 | HIS79 | 3.66, 3.18, 2.99 |
|  | ILE21 | HIS79 | 3.72, 3.64, 3.59, 3.35, 3.26, 3.11, 3.69, 2.98, 3.51, 2.50, 3.43, 2.86, 2.49, 2.02, 2.86, 1.94, 3.20, 2.78 |
|  | GLY22 | HIS79 | 3.17, 3.55, 3.43 |
|  | SER23 | PHE123 | 3.79 |
|  | SER23 | LEU126 | 3.38 |
|  | SER23 | ASP51 | 3.88, 3.48, 3.80, 2.83 |
|  | GLY24 | PHE123 | 3.65, 3.55 |
|  | GLY24 | LEU126 | 3.80 |
|  | GLY25 | PHE123 | 3.85, 3.74, 3.88, 3.31, 3.89 |
|  | GLY25 | MET1 | 3.39, 3.18, 3.59, 3.12, 3.19, 3.87, 2.74, 2.61, 2.87, 3.85 |
|  | GLY25 | ASN2 | 3.83, 2.74 |
|  | PRO94 | LYS78 | 3.88, 2.95 |
|  | GLY95 | LYS78 | 3.86, 3.18, 3.41 |
|  | GLY96 | LYS78 | 3.11, 3.62, 3.30 |
|  | PHE99 | GLY101 | 3.39 |
|  | PHE99 | ALA98 | 3.84 |
|  | ASP100 | ARG72 | 3.41, 3.81 |
|  | ASP100 | LYS78 | 3.65 |
|  | VAL103 | GLN99 | 3.13, 3.60, 3.45, 3.78, 2.63, 2.80, 1.74, 3.75, 2.89 |
|  | VAL103 | ALA100 | 3.32, 3.09, 3.00, 3.51 |
|  | VAL103 | GLY101 | 3.22, 3.82 |
|  | GLU104 | ARG72 | 3.27 |
|  | GLU216 | TYR105 | 2.70, 3.46, 2.14, 3.07 |
|  | GLU216 | ILE94 | 3.07 |
|  | GLU216 | TYR105 | 3.87, 3.41, 2.78, 1.64 |
|  |  |  |  |
| Complex 3 | ASN35 | SER14 | 3.68, 2.89 |
|  | ASN35 | THR15 | 3.83 |
|  | ARG38 | ASP36 | 3.58, 3.56, 3.83, 3.84, 3.14, 3.37, 3.88, 3.64, 3.48, 3.62, 3.88, 3.86, 2.88, 3.85 |
|  | ARG38 | ASP12 | 3.23, 3.69, 3.36, 3.66, 3.81, 2.45, 2.94, 2.72, 2.40, 3.77 |
|  | ARG41 | ASP36 | 3.85, 3.49, 3.78, 3.45, 3.85, 3.64 |
|  | ARG41 | ARG17 | 3.40, 3.25, 3.81, 2.40 |
|  | ARG41 | ASP36 | 3.74, 3.82, 3.72 |
|  | ARG41 | ARG17 | 3.87, 3.62 |
|  | ARG41 | GLU34 | 3.74 |
|  | ARG41 | ALA35 | 3.78, 3.73 |
|  | ASP45 | GLU34 | 3.49, 3.50, 3.42, 3.14 |
|  | ARG48 | THR32 | 3.29, 2.91 |
|  | ARG48 | LYS21 | 3.72, 3.37, 3.15, 3.77 |
|  | ARG48 | GLU34 | 3.66 |
|  | GLY49 | THR32 | 3.38, 3.08, 3.88 |
|  | GLY49 | GLN33 | 3.88, 2.99, 3.60, 3.41 |
|  | GLY49 | ASN31 | 3.57, 3.50, 2.34 |
|  | GLY51 | ASN30 | 3.85, 3.41 |
|  | GLY55 | ARG18 | 3.80 |
|  | ASN56 | ARG18 | 3.83, 3.30, 3.55, 3.23, 3.73, 3.51, 2.81, 3.81, 3.38, 2.41, 3.16, 3.42, 2.17 |
|  | VAL157 | THR40 | 2.65 |
|  | ARG218 | LEU39 | 3.69, 3.04, 3.67 |
|  | ASP365 | ALA73 | 3.75 |
|  | GLN367 | HIS70 | 3.77, 3.77, 3.70, 2.65, 3.53, 3.45, 3.89, 3.53 |
|  | GLN367 | LYS46 | 3.77, 3.68 |
|  | GLN367 | LEU42 | 3.53, 3.40, 3.60, 2.50, 3.61, 2.41, 3.21, 1.26, 3.66, 2.88, 3.82, 1.40, 2.95, 1.78, 2.90, 1.55, 3.48 |
|  | GLN368 | LYS46 | 3.75, 3.77, 3.28 |
|  | GLN368 | ASP74 | 3.86, 3.80, 3.36, 2.77 |
|  | LEU371 | LYS46 | 3.51, 3.72, 3.19, 3.81, 2.30, 2.52, 2.57, 3.05, 1.64, 1.26, 1.18, 3.57, 3.31, 2.11, 3.11, 3.66 |
|  | LEU371 | ASP74 | 3.69 |
